# Anal canal to pubis angle: a novel clinical ultrasound technique for the assessment of the anorectal region

**DOI:** 10.1007/s00192-021-04855-2

**Published:** 2021-07-08

**Authors:** Victoria Asfour, Kayleigh Gibbs, David Wertheim, Giuseppe Alessandro Digesu, Ruwan Fernando, Vik Khullar

**Affiliations:** 1 St Mary’s hospital, Imperial College Healthcare NHS Trust, Paddington, W2 1NY UK; 2 London North West University Healthcare NHS Trust, London, UK; 3 School of Computer Science and Mathematics, Kingston University, Surrey, UK

**Keywords:** Prolapse, Ultrasound, Incontinence, AC to pubis angle, Validation

## Abstract

**Introduction and hypothesis:**

Posterior compartment prolapse is associated with severe morbidity including faecal incontinence and defaecation dysfunction. The aim of this study was to develop and validate a novel ultrasound marker (anal canal to pubis angle) for the assessment of the anal axis in the context of posterior compartment prolapse in women and in controls (healthy, nulliparous, non-pregnant volunteers).

**Methods:**

Anal canal to pubis (AC/Pubis) angle is measured with 2D transperineal ultrasound in precisely the midsagittal plane. The image was inverted and zoomed out and the angle opened to 107° (maximum). The image includes the pubis, urethra and anal canal. The angle measurement starts from the anal canal, pivots on the anorectal junction and ends at the shadow of the pubis. Inter- and intra-observer agreement in AC/Pubis angle measurement was assessed and the angles measured in the two groups compared.

**Results:**

Forty women with posterior prolapse and 17 controls were included. Close agreement was observed in inter- and intra-observer AC/Pubis angle measurements assessed with Bland-Altman analysis. AC/Pubis angle is significantly wider in prolapse patients compared to controls (*t*-test, *p* < 0.001), with mean AC/Pubis angle in prolapse patients 122.9° (SD 15.6°) and controls 98.2° (SD 15.9°).

**Conclusion:**

The AC/Pubis angle is a novel validated 2D ultrasound technique for the assessment of the anorectal axis that potentially can be performed using equipment that is widely available in routine clinical practice. The AC/Pubis angle is significantly wider in prolapse patients compared to controls.

## Introduction

Posterior compartment prolapse and anorectal dysfunction are a challenging area that overlaps urogynaecology and colorectal surgery. The morbidity caused by posterior compartment dysfunction in this area includes defaecation dysfunction and anal incontinence. Several markers and techniques have been proposed in the literature, of which the vast majority of the posterior compartment ultrasound literature focuses on anal sphincter integrity [1–3].

The axis of the anal canal has been assessed with magnetic resonance imaging (MRI) studies in relation to the posterior rectal wall in children with congenital anomalies [4]. An anteriorly displaced anus in children is thought to be a cause of obstructive defaecation. The anal axis has not been assessed in relation to prolapse in women. The anal axis may be different in women with prolapse compared to controls, and it may have a role in the symptom of obstructive defaecation. Anterior to the anal canal is the perineal body. The perineal body is an important structure of the female pelvic floor. The perineal body is smaller in patients with posterior compartment prolapse compared with controls [5].

There is conflicting evidence regarding the association between posterior compartment prolapse and functional bowel symptoms. Obstructed defaecation, particularly the distressing symptom of digitation, has been associated with advanced posterior compartment prolapse [6, 7]. There is no association between POP-Q severity and functional bowel symptoms [8–11].

To make imaging in urogynaecology more accessible and useful to clinicians and their patients, work is needed on developing imaging markers that are practical. Two-dimensional ultrasound scanning technology is widely available and in routine clinical practice in other areas of obstetrics and gynaecology.

The purpose of this study was to develop and validate a new ultrasound marker on 2D trans-perineal ultrasound for the assessment of the pelvic floor: the anal canal to pubis angle (AC/Pubis angle). The angle was applied for the assessment of the anal axis in the context of posterior compartment prolapse in women and in a control group with no prolapse. This study investigates the difference in the AC to pubis angle in control patients and in patients with posterior compartment prolapse.

## Methods

The study was performed with ethical approval by the Riverside Ethics Committee (IRAS 17/LO/1398). Health Research Authority approved the study. Patients with posterior compartment prolapse were recruited from the urogynaecology clinic. A control group of non-pregnant, nulliparous volunteers were recruited from other gynaecology clinics. The inclusion criteria for the control patients were that they needed to be healthy, not pregnant, nulliparous and asymptomatic for urogynaecology complaints such as incontinence and prolapse symptoms. Control patients who were found to have incontinence or prolapse during the assessment were excluded from the study. The prolapse cohort were patients on the waiting list for posterior compartment prolapse surgery. All the patients had a clinical assessment, including POP-Q.

This study was performed with a Voluson E8 ultrasound scanner (GE Healthcare, UK) and using the AB2-7-D (2-7 MHz) probe. The patient was asked to void prior to the scan. The gelled probe was covered with a plastic sheath for hygiene and cleaned between patients. The patient was in the supine, semi-recumbent position with the legs comfortably apart and the prolapse reduced. Table 1 describes the settings of the ultrasound machine that are set prior to the scan. The measurements were conducted with a zoomed out midsagittal trans-perineal 2D image. The image was inverted so that the pelvic organs appear upright.

**Table 1:** Ultrasound machine settings

Probe	AB2-7-D
Application	Gynaecology
Setting	Bladder
Angle	107°
Dynamic control	7
Focal zones	1
Harmonic frequency	MID
XBeam	CRI 3
SRI	SRI 3
Zoom	1.1×
Grey map	4
Depth adjust	13.6
Tint	Clear
Line density	Normal
OTI	Normal
Line filter	Line filter low
Reject	25
Gain	3
Other	Persistence 3 Enhance 1

**Fig. 1:**
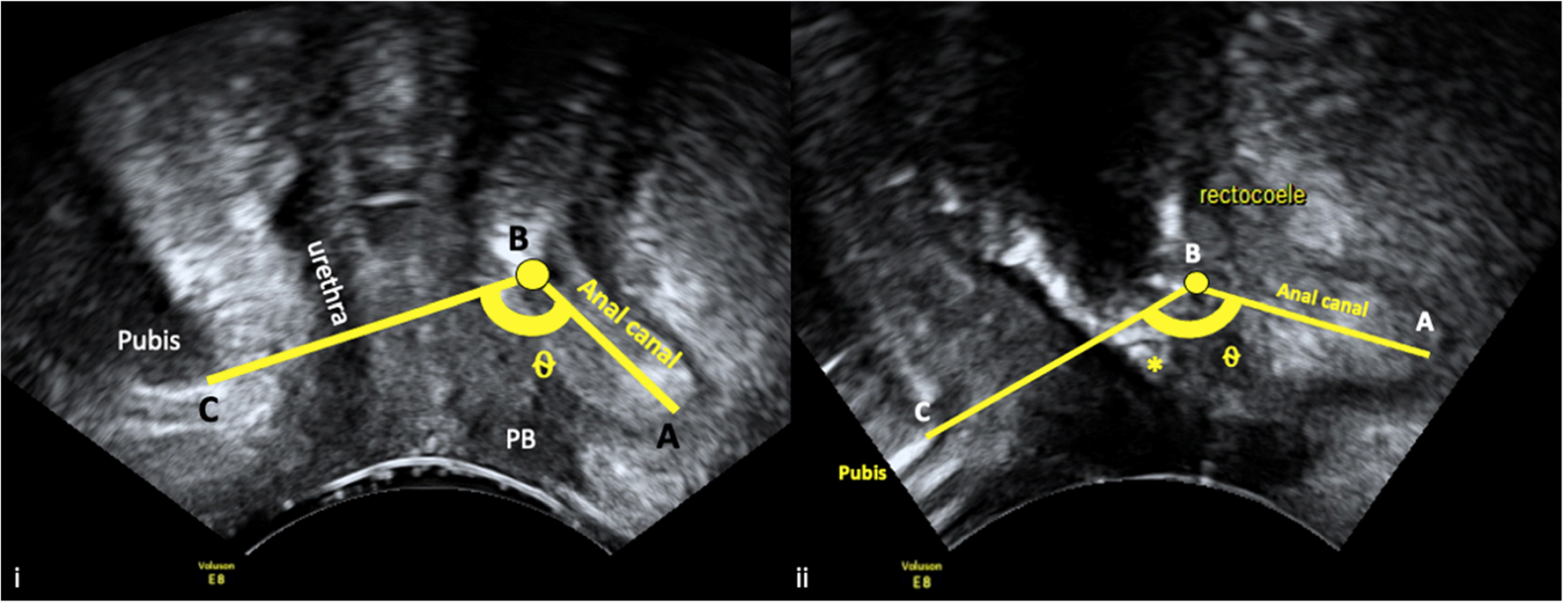
AB is the length of the anal canal. B marks the anorectal junction. BC is the line joining the anorectal angle to the pubis. ⍬ is the AC/Pubis angle. Anterior to the anal canal is the perineal body. The symphysis pubis, urethra, perineal body and anal canal are aligned in the mid-sagittal plane. (i) Anal canal to pubis (AC/Pubis) angle ⍬ in a healthy volunteer. (ii) Anal canal to pubis (AC/Pubis) angle ⍬ in posterior compartment prolapse patient. *Rectocoele

From anterior to posterior, the image was adjusted to include the pubis, urethra, bladder, vagina and anal canal. The anal axis was assessed in relation to the symphysis pubis, which is the most stable structure on the pelvic floor on ultrasound. An angle was measured from the anal canal to the symphysis pubis, pivoting the angle on the anorectal junction. The first line is the midline echo of the anal canal along its length. The first line ends at the anorectal junction. The second line starts at the anorectal junction and finishes at the lower border of the pubic bone; see Fig. 1. This measurement is taken at rest and angles were recorded to two decimal places.

To assess intra-observer repeatability, two angle measurements were taken by the same clinician in live scanning mode separated by a week. To assess inter-observer agreement, the angle of the same image was also measured by another co-author.

Statistical analysis was performed using SPSSv27 (IBM Corp., USA) and Minitab v19 (Minitab Inc., USA). Data were tested for consistency with a normal distribution using the Shapiro-Wilk test in SPSSv27 and parametric or non-parametric statistics used as appropriate. Bland-Altman analysis [12], performed with a macro in Minitab, was used to assess intra-observer repeatability and inter-observer agreement in the measurement of the AC to pubis angle. The Bland-Altman analysis involved plotting the difference between paired measurements against the mean of those paired measurements [12]. The graphs include three horizontal lines, parallel to the x-axis, to indicate the mean difference between the measurements and either side horizonal lines at a position of mean + 1.96 × standard deviation of the differences representing the upper and lower 95% limits of agreement. The inter-observer repeatability and intra-observer agreement for the anal canal to pubis angle measurement were performed for both the control and prolapse patient groups. A comparison of measurements obtained from control and prolapse patients was also performed using a two-sample *t*-test.

## Results

Eighty-six patients were considered in this study. Sixty-seven women with prolapse were studied. Of these, there were 40 women with posterior compartment prolapse (Bp > −3). Twenty-seven women had prolapse in other compartments, but not posterior compartment prolapse (12 had middle compartment prolapse and 15 anterior compartment prolapse); these patients were not included in the analysis in view of the lower number in each group. The control patients were 17 nulliparous non-pregnant young women.

The mean age of the control group was 33.4 years (SD 3.8). The prolapse patients had a mean age of 57.3 years (SD 15.2). The age of the two groups was significantly different (two-sample *t*-test, *p* < 0.001). The Shapiro-Wilk test (*p* = 0.5 in controls and *p* = 0.4 in prolapse patients) showed that AC to pubis angle data are consistent with a normal distribution.

Of the cohort that had Bp > −3 (*n* = 47), 11 had Bp = −2, 12 had Bp = −1, 10 had Bp = 0 and 14 had Bp > 0 (1 to 8).

**Fig. 2:**
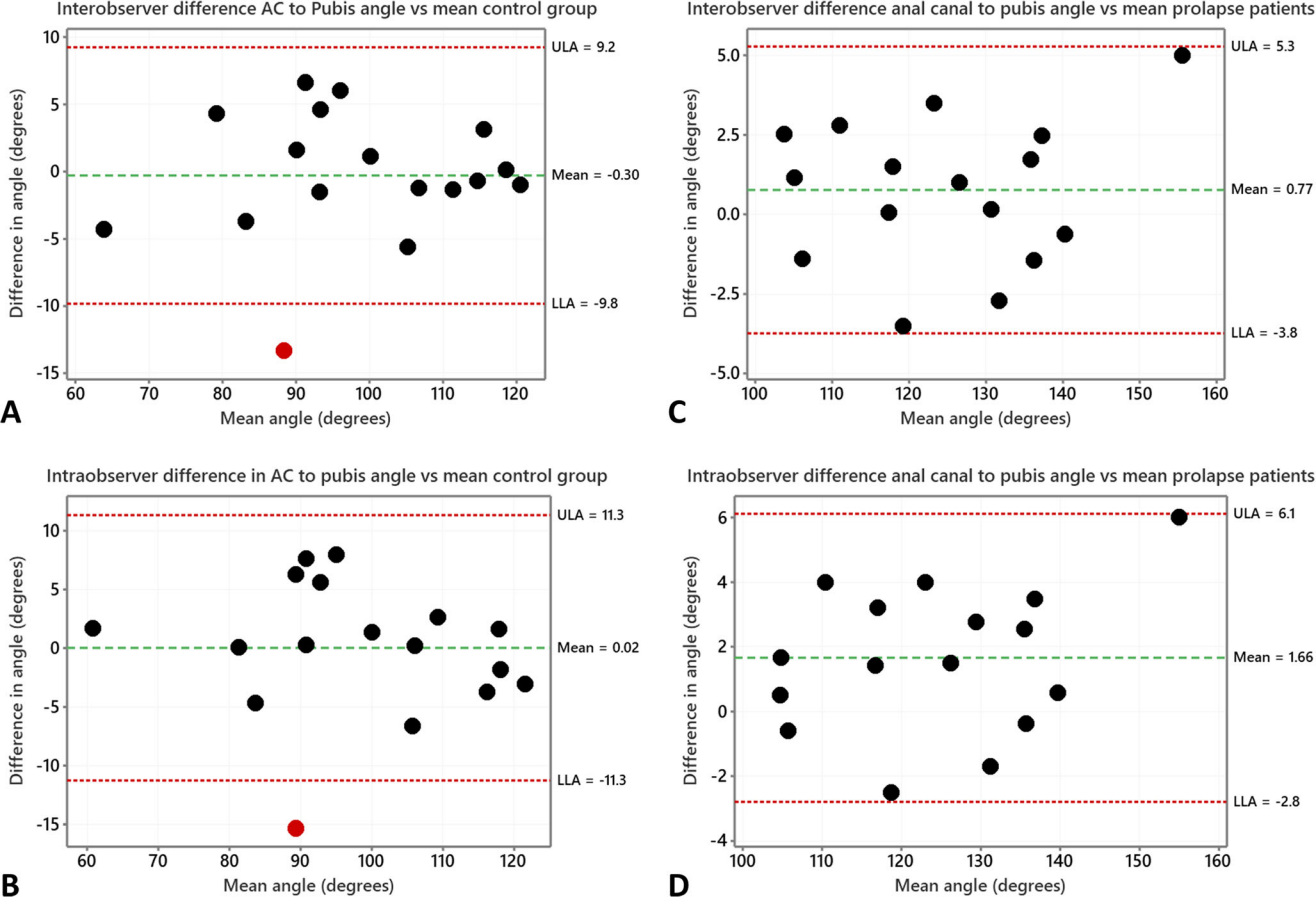
Bland-Altman plots for AC to Pubis angle. The dashed horizontal line parallel to the x-axis indicates the mean difference between the measurements and either side of that line are horizonal lines (dots) at a position of mean + 1.96 × standard deviation of the differences as these represent the lower and upper 95% limits of agreement (LLA and ULA respectively). Points outside of the limits of agreement are shown in red. Graph A shows inter-observer agreement and graph B intra-observer repeatability for control patients. Graph C shows inter-observer agreement and graph D intra-observer repeatability for prolapse patients

Tables 2 and 3 as well as the graphs in Fig. 2 indicate good repeatability in the angle measurements for both control and prolapse patients. In addition, there was very good agreement in the measurements on both groups by the two clinicians with a mean (SD) difference of −0.3° (4.9) and 0.8° (2.3), both of which are clearly smaller than the difference in mean angle seen between the two groups of about 24.7°; there was no significant relation of the differences with the mean angle (*p* = 0.7 and *p* = 0.6) using linear regression indicating that the differences are not dependent on the angle for the range observed.

**Table 2:** Table for inter-observer repeatability and intra-observer agreement for the anal canal to pubis angle measurements in control patients. The mean and standard deviation (SD) were calculated for the individual mean of the measurements as well as the differences. *P* value for difference vs mean from linear regression of difference versus mean

Parameter	*N*	Mean (SD) of measurements (°)	Mean (SD) of differences (°)	Difference vs mean linear regression model *p* value
Anal canal to pubis angle Inter-observer	17	98.30° (15.43)	−0.30 (4.87)	*p* = 0.7
Anal canal to pubis angle Intra-observer	17	98.15° (15.98)	0.02 (5.77)	*p* = 0.6

**Table 3:** Table for inter-observer repeatability and intra-observer agreement for the anal canal to pubis angle measurements in prolapse patients. The mean and standard deviation (SD) were calculated for the individual mean of the measurements as well as the differences. *P* value for difference vs mean from linear regression of difference versus mean

Parameter	**N**	Mean (SD) of measurements (°)	Mean (SD) of differences (°)	Difference vs mean linear regression model *p* value
Anal Canal to Pubis angle Inter-observer	16	124.86 (14.57)	0.77 (2.30)	*p* = 0.6
Anal canal to pubis angle Intra-observer	16	124.41 (14.40)	1.66 (2.27)	*p* = 0.2

AC to pubis angle is significantly wider in prolapse patients compared to controls as seen in Table 4. Prolapse patients have a significantly wider AC/Pubis angle (two sample *t*-test, *p* < 0.001) with the difference of 24.7° between the mean angle in each group. The patient with the narrowest AC/Pubis angle was a young and physically fit nulliparous woman with a strong pelvic floor.

**Table 4:** Anal canal to pubis angle ultrasound measurements in control and prolapse patients. The measurements were compared with a two sample *t*-test. Mean and standard deviation (SD) of the angle measurements are shown as well as number in each group (*N*)

	Mean (SD) in controls (°) *N* = 17	Mean (SD) in prolapse patients (°) *N* = 40	*p* value from two sample *t*-test comparing groups
Anal canal to pubis angle	98.16 (15.87)	122.90 (15.56)	*p* < 0.001

## Discussion

The anal canal to pubis angle is a novel validated measurement for the assessment of the anal axis in the anorectal region. Anal canal to pubis angle was found to be wider in prolapse patients compared to controls (two-sample *t*-test, *p* < 0.001). In this study, the control group were young nulliparous, non-pregnant, asymptomatic women. The prolapse group were peri- and post-menopausal women. Age is known to affect pelvic floor anatomy and functional symptoms [13]. Inherent differences in pelvic floor anatomy have been demonstrated in young versus older nulliparous women without any known or symptomatic prolapse [14]. A longitudinal Irish study of primiparous young women just 1 year after birth showed a 70% (142/202) prevalence of asymptomatic prolapse [15]. Further work in age-matched controls would offer further understanding into the impact of age alone and age with prolapse on the pelvic floor.

When considering all the methods available for imaging the posterior compartment of the pelvic floor at this point in time, MRI or fluoroscopic evacuation proctography is the gold standard of posterior compartment assessment for obstructive defaecation symptoms (ODS) and identification of intussusception [16]. Defaecating imaging involves having a preparation enema followed by barium paste contrast inserted in the rectum [17]. In MRI defaecating proctograms the patient is lying down flat in the MRI scanner and attempts to evacuate the rectal contrast [18]. In fluoroscopic defaecating proctograms the patient needs to evacuate the rectal contrast in the radiography department on a commode during fluoroscopic imaging [17]. These tests have a low acceptability by patients [2].

In echodefaecography, up to 6 cm of the probe is inserted into the rectum [19]. Images are obtained at rest and on Valsalva examining the integrity of the anal sphincter, anismus, presence of rectocoele and intussusception. The endoanal approach was compared with a transvaginal approach [19], finding a near perfect agreement for the presence of intussusception (κ = 0.91) and good agreement for the presence of anismus (κ = 0.76) with these techniques [19].

The data on imaging techniques in relation to intussusception and obstructed defaecation are conflicting. A detailed study of endovaginal ultrasound, transperineal ultrasound, fluoroscopic and MRI evacuation proctograms concluded that there is no optimal scan for the posterior compartment [2]. This study focused on the presence of rectocoele, intussusception, enterocoele and anismus. For example, van Gruting et al. found the sensitivity and specificity of different methods of imaging in detecting intussusception: fluoroscopic evacuation proctogram (sensitivity 60%, specificity 92%, AUC 0.76), endovaginal 3D circumferential (BK) scan (sensitivity 53%, specificity 100%, AUC 0.77), MRI (sensitivity 23%, specificity 100%, AUC 0.62) and transperineal ultrasound (sensitivity 19%, specificity 95%, AUC 0.57) [2], concluding that all the methods have individual benefits but none of the methods is all-round perfect.

A similar comparison was performed by another group, comparing the 3D endovaginal 12-MHz ultrasound with fluoroscopic defaecating proctograms [20]. For detecting intussusception, transvaginal ultrasound had sensitivity 78%, specificity 91%, positive predictive value 56% and negative predictive value 97% and transperineal ultrasound had sensitivity 95%, specificity 92%, positive predictive value 60% and negative predictive value 99%. The authors concluded that defaecatory imaging may be possible to be avoided [20]. Endovaginal circumferential 3D ultrasound can provide good quality imaging, but it is a specialized and expensive resource, limited to centres of excellence.

An Australian group found a poor agreement between trans-labial ultrasound compared to fluoroscopic defaecography for both anorectal angle and rectocoele depth in women with obstructed defaecation [21]. A study on rectovaginal septum thickness was assessed in 3D volumes acquired by the trans-perineal Dietz ultrasound method [22]. The rectovaginal septum thickness measurement was shown to have low repeatability (κ = 0.5) and no clinical significance was found [22]. The paravaginal supports have been studied on 3D volumes at 2-mm intervals, 3 months after vaginal delivery or caesarean section, with no significant anatomical differences found in the two groups [23]. The contractility of the pelvic floor was studied with trans-perineal ultrasound 3D volumes and compared to the clinical Oxford score [24]. Spearman’s correlation between the two groups was moderate (rho = 0.5 for hiatal area and anteroposterior diameter) [24]. There are conflicting data regarding the role of pubovisceral muscle avulsion and faecal incontinence with some studies reporting a correlation [25] but others finding no association [26].

The strength of this work is the development of a novel ultrasound marker for the assessment of the pelvic floor in patients with prolapse and in control patients. Future work in larger cohorts could define a reference range on this marker and further investigate its clinical relevance in urogynaecology.

A limitation of this work is that the prolapse and control cohorts were not age-matched homogeneous groups. Prolapse is an age-related progressive disease affecting up to half of all women in the menopause [27–28]. Ideally, this study would be done on age-matched controls. As prolapse affects mostly older women due to the natural history, we would need to recruit older nulliparous women without prolapse. Parity is a confounding factor that is difficult to control because most women have children. According to American population statistics, only 15% of all women are nulliparous at age 50 [29]. It would be difficult to identify a cohort of age-matched peri- and post-menopausal nulliparous women without prolapse or any other urogynaecological symptoms.

## Conclusion

The anal canal to pubis angle is a novel validated 2D ultrasound measurement of the anal axis that can be performed using equipment that is widely available in routine clinical practice. The angle is larger in posterior compartment prolapse compared to control women.
